# Collaborative clinical facilitation in selected nursing and midwifery colleges in Northern Ghana

**DOI:** 10.4102/hsag.v28i0.2121

**Published:** 2023-03-31

**Authors:** Francis Kobekyaa, Joanne R. Naidoo

**Affiliations:** 1 School of Nursing and Public Health, Faculty of Health Sciences, University of KwaZulu-Natal, Durban, South Africa; 2 Department of Nursing Science, Faculty of Health Sciences, Nelson Mandela University, Gqeberha, South Africa

**Keywords:** clinical learning, clinical mentoring, collaborative clinical facilitation, preceptors, nursing, midwifery training institution

## Abstract

**Background:**

Collaborative clinical facilitation converges key players to guide students individually and within groups towards achieving clinical nursing competence. However, experiences of collaborative clinical facilitation among nurse educators, clinical preceptors and nursing and midwifery students are often fragmented and have been largely unexplored in Ghana.

**Aim:**

To describe the experiences of collaborative clinical facilitation among nurse educators, clinical preceptors and final year nursing and midwifery students in Northern Ghana.

**Setting:**

The study was conducted at two nursing and midwifery colleges and an academic hospital in Northern Ghana.

**Methods:**

A qualitative, descriptive, exploratory design was utilized. Forty-six participants comprising 16 nurse educators, 10 clinical preceptors, 10 nursing students and 10 midwifery students were purposively sampled. Focus groups and in-depth interviews were used to gather data and analysed thematically.

**Results:**

Three themes revealed facilitative experiences of collaborative clinical facilitation: team-based clinical mentorship and supervision, personalised preceptorship, and clinical conferences. Two themes emerged inhibitory to collaborative clinical facilitation: staff shortages and lack of timely communication.

**Conclusion:**

This study found that team mentorship, preceptorship and conferences fostered collaborative clinical partnerships for students’ clinical learning. However, failure to engage in timeous communication in the midst of staff shortages hampered its smooth practice. Orientation workshops need to be organised for key players to share relevant updates and explore ways to navigate the challenges often experienced within the clinical training environment.

**Contribution:**

This paper provides insight into the collaborative nature of clinical facilitation; and highlights the need for coordinated clinical placements to enhance students’ clinical learning.

## Introduction

Collaborative clinical facilitation is fundamental to preparing a well-educated and professionally skilled nursing and midwifery workforce (Clarke, Van der Riet & Bowen 2020). Globally, comprehensive clinical skills acquisition requires collaborative involvement of nurse educators and clinical facilitators or clinical preceptors to guide and mentor students to achieve the requisite skills, knowledge, attitudes and competencies in clinical practice within authentic clinical environments (Mhango 2021; Phuma-Ngaiyaye, Bvumbwe & Chipeta 2017; Rebeiro et al. 2015). Students are guided and supervised individually and/or in groups in their clinical practise. A safe and conducive environment is created for students’ nursing skills acquisition, competencies and standards and integration of theory into practise in the clinical reality of patient care (Grobecker 2016; Stefaniak & Dmoch-Gajzlerska 2020).

Recognising collaborative clinical facilitation as a critical process of ensuring authentic clinical experience, professional nursing councils locally and internationally have prescribed a minimum number of clinical hours for students’ clinical learning competencies at health care facilities (Forber et al. 2015; Gustafsson et al. 2015; Wu et al. 2015). For instance, in Australia a minimum of 800 hours of supervised clinical experience is required, while in the United Kingdom (UK), a minimum of 2 300 clinical hours are needed for professional registration (Forber et al. 2015; Rebeiro et al. 2017). In the African context, South Africa in particular, a nursing or midwifery student is required to complete a minimum of 4 000 hours in clinical placements as part of the requirements for the completion of training as a professional registered general nurses with the South African Nursing Council (Nurse 1985), whereas in Ghana 1 632 clinical hours are required for licensing as a professional nurse (Nursing and Midwifery Council of Ghana [NMCG] 2015).

However, the controversy between the academia and clinical practise on who has the prime responsibility for the clinical teaching of students is a major barrier to the practise of collaborative clinical facilitation (Ford et al. 2016; Jamshidi et al. 2016; Mbakaya et al. 2020). The two main models in use are preceptor and facilitator. The preceptor model involves teaching, role modelling and supporting students to acquire nursing skills and personal attributes in the clinical setting (Dobrowolska et al. 2016; Hilli et al. 2014), whereas facilitation model provides a direct or indirect supervision of a group of students by a facilitator (Franklin 2013). While a facilitator model is preferred over preceptor model, there are varied views on who should facilitate the clinical skills acquisition of students (Ford et al. 2016). For instance, researchers (Anarado, Agu & Nwonu 2016; Atakro & Gross 2016) have noticed that nurse clinicians in practise regard nurse educators as intruders or strangers in health care facilities during student clinical supervision instead of equal professional co-workers. This may be due to the fact that clinical preceptors often see the nurse educators to be completely theoretical and have lost touch with the reality of the clinical setting (Zyl 2014). Franklin (2013) argues that due to the competing demands of academic teaching, supervision and administrative responsibilities of the nurse educator, the role of clinical facilitator is not best suited for them (Franklin 2013). Yet, evidence also shows that the increasing clinical workloads and lack of preceptor training makes the nurse clinicians or clinical preceptors in practise less suited as clinical facilitators (Sundler et al. 2019).

## Purpose of the study

This study aimed at describing the experiences of collaborative clinical facilitation among nurse educators, clinical preceptors, and final year nursing and midwifery students from selected nursing institutions in northern Ghana.

### Research design

This study was underpinned through constructivism that enabled the researcher to construct meaning regarding collaborative clinical facilitation through multiple realities expressed from the nursing and midwifery students, nurse educators and preceptors’ experiences. A qualitative descriptive exploratory design using focus group discussions (FGDs) and individual interviews was used for the study and provided an opportunity for the researchers to be deeply immersed in the inquiry and gained an in-depth understanding of the phenomenon as experienced by the participants (Katowa-Mukwato et al. 2014).

### Research setting

The study was conducted at two Nursing Education Institutions (NEIs) in northern Ghana. The two NEIs provided training towards a Diploma in General Nursing and training in a Diploma in Midwifery, respectively. College A had a total population of 480 nursing students, consisting of 170 students in the 1st year, 160 students in the 2nd year, and 150 students in the 3rd (final) year of study. College B had a total population of 235 midwifery students, which comprised 85 students in the 1st year, 80 students in the 2nd year, and 70 students in 3rd (final) year. Both colleges had a total of 8 and 10 nurse educators, respectively, involved in the theoretical and clinical training of the nursing and midwifery programme.

The selected hospital is a 200-bed referral hospital, which at the time of data collection served a population of approximately 101 000 people living within and outside the local region or municipality zoned for referral to the hospital. Fourteen Primary Health Care (PHC) and Community Health Care (CHC) centres are also referral points of care for the selected hospital. The hospital is a training facility accredited with the two NEIs wherein nursing and midwifery students conduct work-integrated clinical learning.

### Population and sampling

The target population for this study was final year (3rd year) nursing and midwifery students, nurse educators involved in the theoretical and clinical education of the 3rd year nursing and midwifery programme from the two selected NEIs and the clinical preceptors involved in providing clinical facilitation in the selected hospital. Within the context of this study, clinical preceptors referred to professional nurses who served as clinical mentors and accompany the nursing and midwifery students from the selected nursing and midwifery colleges in the clinical work setting. The clinical preceptor provides clinical facilitation for the nursing and midwifery students during their clinical work placements. Part of the clinical facilitation activities includes assessment of clinical competencies against nursing and midwifery programme objectives, as well as facilitating the application of theoretical nursing and midwifery objectives in the clinical and practical work settings. Purposive sampling was used to ensure that participants with the relevant experience were selected.

### Data gathering

Following ethical approval of the study from the Humanities and Social Sciences Research Ethics Committee from a higher education institution in South Africa where the first author was undertaking a postgraduate programme, gatekeeper permission was received from the Head of Department from the two selected Nursing colleges and hospital manager in northern Ghana. Potential participants were contacted through information posters placed at the hospital and the selected nursing colleges. Student information boards were also used to share information about the study, and students could contact the researcher directly. The purpose of the study was explained to participants through an information session and also through an information letter conducted in English. Written informed consent was received from participants who voluntary agreed to participate in the study.

Prior to each data gathering session, the overall aim of the study was verbally explained, and participants were reminded of their right to withdraw from the study at any time without consequence. Data gathering took place onsite either at the selected hospital or nursing college in rooms that had no disturbances. A digital recorder was used to record the FGDs and interviews. Each FGD lasted between 90 and 120 mins, and the individual interviews lasted between 50 and 60 mins. At the point of five FGDs, two were conducted among participants at college A and three were conducted among participants at college B, and four individual interviews were conducted among participants at the selected hospital, no new information contributing to the aggregated themes emerged, and data saturation was established at this point. The central questions for the nursing and midwifery students were:

Kindly describe your experience of the clinical facilitation conducted by the nurse educators and clinical preceptors during your clinical placement and accompaniment? Please describe any challenges and any highlights you have experienced through the clinical accompaniment conducted by your nurse educators and clinical preceptors?

While the central questions for the nurse educators and clinical facilitators were:

What is your current experience of collaborative clinical facilitation? How do you integrate the theoretical and clinical component of the programme in your clinical facilitation? What are your current practices towards collaborative clinical facilitation? What do you perceive to be the barriers to collaborative clinical facilitation? What do you perceive to be the facilitators of collaborative clinical facilitation?

Data gathering occurred over 3 months (i.e. October–December 2016); all information was gathered by the first author and all the FGDs and interviews were conducted in English.

### Data analysis

The multiple approach of collecting information from multiple perspectives of the nursing and midwifery students, nurse educators, and clinical preceptors allowed for a comprehensive understanding of collaborative clinical facilitation. Thematic analysis by Braun and Clarke (2006) guided the data analysis. Verbatim transcription was conducted on all individual interviews and FGDs. This inductive process enabled patterns, categories and themes to emerge through the organisation of the data into units of information or meaning. This was enabled by reading each transcript several times and through line-by-line coding guided by the aim of the study. The units of meaning (codes) were grouped manually into similar concepts and constructs that reflected the experiences related to collaborative clinical facilitation. This enabled the emergence of themes being identified that were categorised into experiences that facilitated and inhibited collaborative clinical facilitation as experienced by nursing and midwifery students, nurse educators, and clinical preceptors.

### Trustworthiness

Trustworthiness was ensured through the application of the four criteria outlined by Lincoln and Guba (1985) namely, confirmability, dependability, transferability, and credibility. Through verification of the collated themes and meaning emerging from the study by the participants allowed for confirmability of the findings. Dependability was ensured through peer review of all the transcripts and data coding and data findings by the second author. Transferability was ensured through a broad description of the study methods, setting, sampling and findings in relation to the context of the study (Mabuza et al. 2014). Credibility of the findings was also ensured through the triangulation of data from the FGDs, individual interviews, and reflections of the researcher during data collection through fieldnotes.

### Ethical considerations

Ethical approval for the study was granted by Humanities and Social Sciences Research Ethic Committee of a selected South African Higher Education Institution (HSS/1553/016M) and the two selected nursing colleges and hospital in Ghana (JH/10 and UWR/JMTC/F). Gate keepers’ permission was also sought from the heads of the three institutions prior to data collection. Participants were informed about the nature and purpose of the study and researchers responded to questions requiring clarification regarding the study before obtaining written consent from each participant. Confidentiality and anonymity were ensured by assigning identifiable pseudonym to each participant; known only to the participants and researchers. The study procedure did not cause any psychological distress or harmful effect to participants.

### Results of the study

The sample composed of 20 final year nursing and midwifery students (i.e. 10 nursing students from college A and 10 midwifery students from college B), 16 nurse educators (i.e. 8 from college A and B, respectively) and 10 clinical preceptors from the hospital. The nursing and midwifery students were between 20–30 years with an average of 23 years, and 12 of the 20 students were female. Of the 16 nurse educators, the age ranged from 26 to 45 years with an average age of 35 years. Regarding work experience, nurse educators had an average of 4 years nursing education experience ranging from 1 to 10 years, the Bachelor of Science (nursing) was the highest qualification for all nurse educators save for one who had completed a Masters in Sciences (nursing) qualification. The age range of the clinical preceptors ranged from 25 to 46 years with an average age of 36 years, and the highest qualification achieved was a post-basic diploma in general nursing or midwifery, with an average of 5 years clinical experience.

Five themes related to the facilitative and inhibitory experiences of collaborative clinical facilitation included: (1) ‘team-based clinical mentorship and supervision’ and (2) personalised preceptorship and (3) ‘clinical conferences’. Themes as inhibitory to collaborative clinical facilitation included: (4) ‘staff shortages’ and (5) ‘lack of timely communication’ between healthcare facilities and nursing and midwifery colleges.

#### Team-based clinical mentorship and supervision

Collaborative clinical facilitation was experienced by participants as a form of team-based clinical mentorship characterised by joint facilitation and supervision of students. Clinical preceptors expressed that clinical mentorship supported students in navigating new clinical skills and in challenging situations. Nursing students reflected that their experience of clinical facilitation allowed them to interact with other practitioners and learn from various professional nurses in the clinical setting, which allows them to further harness their clinical skills. This is noted in the excerpts:

‘Joint supervision and mentorship is one of the activities that eeehh … we undertake to jointly facilitate, guide and supervise students as they carry out their clinical activities. We also, together with the other nurses, mentor students’ learning to provide quality nursing care and to ensure patients’ safety during difficult clinical situations.’ (P2, Female, Clinical Preceptor)

‘During clinical supervision, all staff help us to improve upon our clinical skills, as well as interact with experienced nurses.’ (T2, Male, Nursing Student)

Nurse educators are professionally bound to provide support to students in collaboration with other role players and as such regarded collaborative clinical facilitation as a mandatory regulatory requirement for professional nurses to carry out in order to gain credit points for renewal of the Professional Identification Numbers (PINs) with the NMCG:

‘I will say, clinical facilitation is done while they [*students*] are in the ward, because it is also part of errrh … our prerogative or regulatory requirement that we supervise and guide students in the ward to be capable of meeting the health needs of the people, and then, we use the credit points for the renewal of our PINs as instructed by NMC.’ (T4, Male, Nurse Educator)

Maintenance of clinical currency of nurse educators through collaborative clinical facilitation was acknowledged. Nurse educators felt that provision of guided supervision to students helped them to spend some time in the ward to update their skills in clinical practise, as they were out of the ward as educators:

‘And also during the clinical supervision, we do monitoring and I also get the opportunity to spend some time with the students in the wards and you update yourself with contemporary clinical skills.’ (T4, Male, Nurse Educator)

Moreover, clinical preceptors experienced collaborative clinical facilitation as a platform that allowed all role players to connect and engage in peer supported learning and information exchange. The excerpts below express these views further:

‘During clinical supervision and mentoring, nurse educators and preceptors come to join us [*and*] share ideas to help in their clinical learning. And also, as you supervise, you also get the opportunity to learn from students and other staff on contemporary nursing practice.’ (T5, Female, Clinical Preceptor)

According to the preceptors, direct involvement and physical presence of the staff supported students to acquire the needed skills and nursing competencies for professional practice upon completion as shown in the following excerpt:

‘What we do is that we accompany students to the clinical grounds or to the wards … and then we form a team that comprises of the preceptors, the nurse educators and the nursing staff, together with the students to support them [*students*] to acquire skills for practice.’ (P1, Female, Clinical Preceptor)

The students found that the clinical supervision helped in them developing professional identify through the socialisation with the clinical preceptors. The students also indicated that they received adequate support during clinical supervision, and they expressed an increase in their self-efficacy and self-esteem in clinical practice due to the support received. This is reflected in the following selected excerpts:

‘With my experience, when the tutors, preceptors and nurses come to supervise us, they try to tell [*us*] how to behave in the ward as future nurses and midwives … we also try to model our behavior to what they are teaching us.’ (T3, Female, Midwifery Student)

‘When staff comes to supervise us, they boost our confidence … We are no more scared of anything.’ (T2, Male, Nursing Student)

The excerpts and reflections within this theme provided a description of the experiences of clinical facilitation as being a space where collaborative learning took place, which provided support and guidance through a team-based approach facilitated by the nurse educators and clinical preceptors.

#### Personalised preceptorship

Personalised preceptorship emerged as form of collaborative clinical facilitation. Nursing students observed close and effective preceptorship during their clinical practise, where there was one-on-one interaction with an experienced professional nurses who were also appointed as clinical preceptors within the nursing and midwifery programme selected in this study. The clinical preceptors who were professional nurses in the clinical settings provided support, clinical mentoring throughout the students training, and preparation for clinical practical exams. This was experienced as an enabling attribute of the collaborative clinical facilitation, as the nursing and midwifery students expressed that the clinical preceptors and nursing educators provided individual support and clinical supervision, which was helpful in the clinical exam preparation periods:

‘There is always one-on-one interaction with a professional nurse at the ward. They [*staff*] do also close supervision of us during exams.’ (T2, Female, Nursing Student)

‘During clinical practice at the ward, we [*students*] get the opportunity to be with professional nurses in the wards, and we carry out procedures with their [*staff*] assistance, it helps us to learn directly from the professional nurses so we don’t cause harm to the patients.’ (T8, Female, Midwifery Student)

‘When we are with the nurses … they are experienced, so they share their skills with us, sometimes individually and other times in a group, and that helps us to develop clinical skills so that we can practice well when we complete.’ (T9, Male, Nursing Student)

According to the preceptors, students were paired with experienced professional nurses, either individually or in groups, to engage in clinical practise which ensured effective professional modelling and socialisation. In other words, the one-on-one support and guidance given to students in the ward helped them gain deeper appreciation and understanding of the responsibilities in the nursing and midwifery professions. Further to this, clinical preceptors expressed similar views, stating that the one-on-one interaction between an experienced practitioner and a student supported and motivated the students to learn. The excerpts below highlight these views further:

‘So as preceptors we do some pairing with students. It gives us the opportunity to be with the students at the clinical site so that they are properly modeled.’ (P3, Female, Clinical Preceptor)

‘Again, the one-on-one supervision given to students makes them understand the responsibilities in the nursing profession.’ (P4, Female, Clinical Preceptor)

‘The one-on-one support we give to students motivates them to put up their best and that is what we do.’ (P5, Female, Clinical Preceptor)

Reflected in this theme, participants described the value the nursing and midwifery students experienced in having clinical preceptors who were professional nurses from the clinical settings. The students expressed that this enabled them to learn directly from the clinical preceptors through direct observation of clinical skills. Moreover, the individual clinical mentoring served as a support for clinical practical exam preparation by the students.

#### Clinical conferences

Recognised as a means of gathering all key stakeholders together to preview and review the clinical activities of the students, preceptors considered clinical conferences as an integral space to engage in a more collaborative manner with other stakeholders. The clinical preceptors indicated that within the clinical conferences, nursing and midwifery students had an opportunity to interact and learn from other professionals involved in the clinical ward or clinical training space. Thus, the knowledge and skills of students were augmented by the clinical staff to obtain a complete understanding of their experiences in the practise settings:

‘It is the only platform [*clinical conference*] that I see students directly interacting with all the staff at one place and to discuss about clinical activities.’ (P4, Female, Clinical Preceptor)

‘We do see the other staff coming to join in the meeting with the students and [*us*], the preceptors. And in the meeting, we add to the knowledge and skills students are lacking, for them to have complete understanding.’ (P3, Female, Clinical Preceptor)

It further emerged from the data that nursing and midwifery students believed that clinical conferences provided safe platforms for them to express their feelings and concerns and challenges with the other key players. Further to this, one of the student participants in the focus groups recalled fondly the great times they had during clinical conferences. Some, however, revealed that they did not get the opportunity to witness or experience clinical conferences as a result of staff shortages, as they were always busy in the ward and did not have much time for clinical conferences. The excerpts below express these views further:

‘Clinical conferences are good times; we share our feelings about the attachment and the challenges we face with the others.’ (T2, Male, Nursing Student)

‘in the clinical conference so she [*ward in-charge*] will mention your name and then ask you what did you do or what is your experience? So, you share it with the whole group and I think it was always interesting. We all enjoy it.’ (T3, Female, Midwifery Student)

Reflected in this theme, the clinical conferences were organised as feedback sessions following clinical period’s activities for students to interact, share and learn from each other’s clinical learning experiences. This also allowed for peer learning and reflection on their clinical learning experiences with their colleagues and the clinical teaching team.

#### Staff shortages

The shortage of staff emerged as a barrier to collaborative clinical facilitation. Participants noted that staff at the hospitals in particular were few given the number of students on clinical placement requiring clinical teaching and support. For instance, clinical preceptors expressed dissatisfaction about their limited numbers compared to the large number of students requiring clinical mentoring and support. As a consequence, clinical preceptors indicated that they were not able to guide and facilitate students’ learning on a one-to-one basis at all times:

‘We are not many and [*the*] students’ population has increased, and when students are sent to the various wards, ultimately we will not be available to really facilitate at all times.’ (P2, Female, Clinical Preceptor)

‘But the preceptors are just [*a*] few. You have a school with over 500 students, and we have three preceptors … The inadequate [*number of*] preceptors is one other challenge.’ (P4, Female, Clinical Preceptor)

Additionally, the clinical preceptors expressed feeling overwhelmed with the dual responsibilities of providing direct patient care provision and facilitating clinical learning for the nursing and midwifery students, especially in instances when there were many students. This is reflected in the following excerpt:

‘We are few and we can’t be caring for patients and teaching students also.’ (T5, Female, Clinical Preceptor)

The nurse educators also shared challenges related to staff shortages and described that, as nurse educators, the competing demands of other academic responsibilities in the college and the number of students in the programmes made it difficult to visit the clinical facilities to supervise and guide students’ clinical learning:

‘Then again, we the tutors [*nurse educators*], we are not also enough. You have one tutor taking more than two courses. For instance, a tutor taking Advanced Nursing and at the same time Basic Nursing and handling a class of about 200 students or 170 or 150 students. So, it puts pressure on us to join the other staff to supervise students.’ (T1, Male, Nurse Educator)

‘The tutors, also some of them are not best-suited for clinical facilitation because some are not nurses [*they are Disease Control; and Information Technology Tutors*].’ (T4, Female, Nurse Educator)

Moreover, the nursing and midwifery students expressed an implication of the staff shortages, and/or limited clinical preceptors did add to frustrations experienced by students, especially during periods of clinical exam preparation where students felt they required more clinical mentoring and supervision by their clinical preceptors. Students also noted that clinical preceptors may displace their frustrations on the students due to the demands experienced by the clinical preceptors:

‘… they don’t supervise us all the times because they are so busy attending to patients and doing other things.’ (T9, Male, Nursing Student)

‘And so because the nurses are inadequate with so much workload, at the end of the day they overwork, get frustrated and infuriated and when it happens like that they displaced their anger on us, the students ….’ (T8, Female, Midwifery Student)

The experiences relating to staff shortages were noted within the nursing colleges, and the clinical setting and participants noted the challenges experienced in terms of being available to adequately spend time with the students in harnessing their clinical skills and experiences.

#### Lack of timely communication

Poor coordination of critical information related to the nursing and midwifery programme was cited as a challenge in terms of the feedback and communication between the clinical training facility (i.e. the hospital) and the nursing education institutions (i.e. the colleges). The participants also expressed an acknowledgement of the important role timeous information sharing is in terms of facilitating the achievement of the clinical learning objectives inherent in the nursing and midwifery programme:

‘We don’t share information among ourselves and we do not communicate effectively, so we tend to repeat certain mistakes.’ (P2, Female, Clinical Preceptor)

‘… we don’t share information timely regarding students clinical placements, but it is important we do so in order to build a stronger clinical team for students’ learning.’ (T5, Male, Nurse Educator)

Participants expressed incidents wherein the lack of timely communication resulted in poor coordination and missed clinical learning time for the students, especially in instances where the clinical learning facilities were not informed of the student’s placement in a particular ward to accommodate allocation of the students. Communication in respect of the student’s role of not wholly or poorly communicating the expected clinical learning objectives that are required to being fulfilled was also noted:

‘The colleges send students to the ward without giving hospital nursing leadership early notice. Students don’t also bring learning objectives to the ward as a guide for us to support them.’ (T5, Female, Clinical Preceptor)

These reflections of the nurse educators lend support to that of the preceptors presented above. The nurse educators reported that students were once sent to the health care facility for placement and were returned to the colleges because college management did not give timeous information to the hospital nursing leadership to prepare for students’ clinical placements:

‘Our students were once returned to the school because management of our school did not give prior information to the hospital nursing leaders to prepare for the students.’ (T1, Male, Nurse Educator)

Reflected in this theme, the clinical preceptors and nurse educators expressed the consequence of the lack of timely and effective communication among the key players in ensuring effective guidance and supervision of students’ placement learning experiences. Despite the lack of timely information sharing at the institutional level and between the leadership of the nursing education institutions and the clinical learning facility, the role-players who were operationally involved in the students’ clinical learning; namely the clinical preceptors and nurse educators expressed ways in which they attempted to mitigate the information as void and not compromise the clinical learning of the students.

## Discussion

This study provides insight into the experiences of collaborative clinical facilitation among nurse educators, clinical preceptors, and nursing and midwifery students. The study found collaborative clinical facilitation as a platform for team-based clinical mentorship and supervision where students receive guided and supervised practise on patients in the clinical learning environment. These findings were in line with the views of many researchers (Antohe et al. 2016; Kristofferzon et al. 2013; Mbakaya et al. 2020), who emphasised that nurse educators, preceptors and clinical nurses were obliged under the scope of their practise to provide daily clinical supervision to nursing and midwifery students by guiding, mentoring and socialising them into the nursing and midwifery profession. This was crucial because these key role players were expected to carry out supervision in order to demonstrate how the classroom theoretical knowledge was applied into real-life clinical nursing situations, for students to acquire skills and competencies in clinical training (Crecious, Patricia & Faston 2018; Dehghani et al. 2016). For Antohe et al. (2016), the overall intent and purpose of joint supervision and, for that matter, team-based mentoring and supervision, was to provide a balance between academic and practical learning, showing practical skills in clinical practice.

Preceptors stated that they carried out clinical mentorship and supervision to collaboratively offer students guidance and support during clinical practice to ensure patients’ protection and safety during difficult clinical situations, as advocated by Phuma-Ngaiyaye et al. (2017) and Mhango (2021). This also helped nursing and midwifery students to develop better relationships and communication with the nurse educators, clinical nurses, preceptors and with each other (Dehghani et al. 2016).

Furthermore, personalised preceptorship surfaced as an important approach to collaborative clinical facilitation. According to nursing students, preceptorship was experienced as close supervision characterised by one-on-one interaction with a professional nurse in the practise setting. Several studies have documented similar findings (Asirifi et al. 2013; Crecious et al. 2018; Phuma-Ngaiyaye et al. 2017; Walker et al. 2013) advocating that, for a pre-determined period, the student must be paired with an experienced practitioner on a one-on-one basis, with both working at the same shift and the preceptor providing direct evaluative feedback to the student.

Consistent with other studies (Flott 2017; Vezeau 2016), this study found that clinical conferences were recognised as an important means of gathering all key players together to preview and review the clinical activities of nursing students. Reflected in this study, the participants noted that the clinical conferences were the only media through which students got engaged in a more collaborative manner with all stakeholders. Students got the opportunity to interact and learn from each other. Thus, the knowledge and skills of students were augmented by staff, to obtain a complete understanding of their experiences in the practise settings as observed by Vezeau (2016).

Chief among challenges confronting collaborative clinical facilitation as experienced by participants, was shortage of staff at the health training institutions and healthcare facilities. Participants reported few staff present compared to the large number of student nurses and midwives available for facilitation, and this was similar to the findings by Msiska, Smith and Fawcett (2014). Atakro and Gross (2016) indicated that due to a shortage of staff, it was very common to have more students than staff assigned to a ward and therefore not able to give personalised attention to every student. Consistent with this study, preceptors in this study explained that due to their limited number, they were not available at each shift to facilitate students’ learning on a one-to-one basis, as mandated by their scope of practise. In effect, staff shortages make clinical learning experiences very difficult, risking the student graduating with skills deficits (Msiska et al. 2014).

Consistent with other studies (Mbakaya et al. 2020; Malwela et al. 2016; Phuma-Ngaiyaye et al. 2017), preceptors explained that the challenge of infrequent and lack of timely information exchange between healthcare facilities and nursing/midwifery colleges was manifested through errors repeatedly committed and inconsistencies in the organisation of clinical placements. For Forber et al. (2015), the reason has been the shift in nursing education from the hospital-based apprentice training of the past to higher-level preparation, which invariably resulted in the ‘uncoupling’ of health facilities and nursing colleges, with a clear lack of sustained and timely communication between them during clinical placements. Nurse educators in this study cited instances where students sent to hospitals for placements were returned to the nursing or midwifery colleges because managements of the colleges did not give timely information to the hospital nursing leadership to prepare for the students’ placement. Meanwhile, timely and sustained communication between hospitals and colleges’ managements is a key requirement for effective students’ placement in clinical practise (Dehghani et al. 2016).

Additionally, clinical preceptors lamented that students came to the health facilities for placement without learning objectives as a guide for their training. This was congruent with Stefaniak and Dmoch-Gajzlerska (2020) findings where lack of timely exchange of information between staff of nursing institutions and health facilities on the learning objectives of the students hindered the provision of efficient clinical facilitation. Consequently, clinical staff and preceptors felt reluctant to perform their clinical teaching role, which impacted negatively on the students’ placement learning experiences (Malwela et al. 2016; Stefaniak & Dmoch-Gajzlerska 2020).

## Conclusion

Collaborative clinical facilitation is at the centre of quality clinical learning experiences for students across several disciplines particularly nursing and midwifery worldwide. It is a useful tool that provides a converging point for key players to guide students either individually or in groups to acquire nursing skills, competencies and standards for patient care. In this study, participants experienced collaborative clinical facilitation as forms of team-based clinical mentorship and supervision, personalised preceptorship and clinical conferences. It also emerged from this study that lack of timely communication between healthcare facilities and nursing educational institutions as well as staff shortages affected the effective operation of collaborative clinical facilitation. These constraints imply that there is the need to resource these institutions in order to optimise students’ placement learning experiences. Further, placement orientation workshops and clinical accompaniment practises must be created to allow both nurse educators and clinical preceptors to connect, ideate and share relevant updates on the latest developments in the nursing curriculum and clinical practise. However, it also emerged from the study that lack of coordination and collaboration between healthcare facilities and nursing educational institutions as well as shortage of staff affected the effective operation of collaborative clinical facilitation. These constraints imply that there is the need to resource these institutions in order to strengthen collaborative partnerships between healthcare facilities and nursing and midwifery colleges. More importantly, the role of the nurse educator and the clinician needs to be incorporated into the students’ clinical rotations programme aimed at providing a coordinated and organised clinical learning environment for an effective collaborative clinical facilitation.
